# Laplacian Eigenfunctions Learn Population Structure

**DOI:** 10.1371/journal.pone.0007928

**Published:** 2009-12-01

**Authors:** Jun Zhang, Partha Niyogi, Mary Sara McPeek

**Affiliations:** 1 Department of Radiology, The University of Chicago, Chicago, Illinois, United States of America; 2 Departments of Statistics and Computer Science, The University of Chicago, Chicago, Illinois, United States of America; 3 Departments of Statistics and Human Genetics, The University of Chicago, Chicago, Illinois, United States of America; University of Utah, United States of America

## Abstract

Principal components analysis has been used for decades to summarize genetic variation across geographic regions and to infer population migration history. More recently, with the advent of genome-wide association studies of complex traits, it has become a commonly-used tool for detection and correction of confounding due to population structure. However, principal components are generally sensitive to outliers. Recently there has also been concern about its interpretation. Motivated from geometric learning, we describe a method based on spectral graph theory. Regarding each study subject as a node with suitably defined weights for its edges to close neighbors, one can form a weighted graph. We suggest using the spectrum of the associated graph Laplacian operator, namely, Laplacian eigenfunctions, to infer population structure. In simulations and real data on a ring species of birds, Laplacian eigenfunctions reveal more meaningful and less noisy structure of the underlying population, compared with principal components. The proposed approach is simple and computationally fast. It is expected to become a promising and basic method for population genetics and disease association studies.

## Introduction

Principal Components Analysis (PCA) is a classical statistical tool to achieve dimension reduction through consideration of linear combinations of the original variables. The top few principal components (PCs) are the linear combinations that explain the greatest amount of variation in the data. The use of PCA in population genetics has a long history, including early work of Cavalli-Sforza and colleagues [1], [2], who considered high dimensional genetic variants from population samples at many different continental locations and used the top PCs to summarize the genetic variation across space. While legitimate concerns have been raised about the interpretation of such PC maps [3], PCA can still provide useful information and is a commonly-used tool in various contexts of genetic data analysis [4]. For example, there is known to be a close connection between the spectral decomposition of the migration matrix and that of the genetic covariance matrix [5]. More recently, in genome-wide disease association studies, PCA has been employed to detect and correct population stratification [6]–[8], in which systematic ancestry differences between cases and controls can lead to false positive association between phenotype and genotype. Such spurious associations [9]–[11] can occur when the disease frequency varies across subpopulations, resulting in affected individuals being more likely than unaffected individuals to be sampled from certain subpopulations [12]. Though this topic has been extensively studied, PCA has advantages [6] over other methods such as genomic control [13] and structured association [14].

Motivated from geometric learning [15], we describe LAPSTRUCT, a Laplacian eigenfunction approach based on graph theory which we briefly introduced in Genetic Analysis Workshop (GAW) 16 [16]. One regards each subject as a vertex of a weighted graph [17], where the weight associated to the edge for each pair of subjects is chosen as a function of their genetic relatedness, with higher weight given when individuals are genetically closer (see Methods). Thus, in this context, one thinks of the distance between each pair of subjects as being based on their degree of genetic relatedness, not on their geographical proximity. The resulting adjacency graph approximates the underlying manifold of the dependence structure of the sample. The eigenfunctions of the Laplace-Beltrami operator [18] on the manifold are generalized geometric harmonic functions, which contain useful intrinsic geometric structure information on the population. The eigenvectors of the associated graph Laplacian matrix (see **Methods**) are first-order linear approximations of the Laplacian eigenfunctions, and they relate to the intrinsic dependence structure of the data. The Laplacian eigenmap formed by embedding each subject to a lower dimensional Euclidean space via the top few eigenfunctions has a locality preserving property, that is, the distance between a pair of subjects in the Laplacian eigenmap reflects the degree of their being correlated. The more they are correlated, the closer together they are mapped. As a result, the Laplacian eigenmap leads to cluster-like structures for subjects who either come from the same discrete subpopulation or share more common ancestry in an admixed population.

The Laplacian eigenfunction method is part of a large class of spectral methods that includes PCA as a special case. However, the approach we use improves on PCA in that each vertex is connected by edges to only its close neighbors, rather than to all other individuals (where, here, closeness refers to genetic relatedness rather than physical proximity). A justification for this results from the connection between spectral clustering and approximate solutions to graph cut problems (see previous work [19], [20] for details). The result is that the Laplacian eigenfunction method tends to emphasize substructure that affects many data points rather than just a few extreme points, so the proposed nonlinear algorithm is robust to outliers, in contrast to PCA. Therefore we suggest using Laplacian eigenvectors instead of PCs to study population structure. A similar approach based on spectral graph theory is also treated by Lee et al. [20] with a nice illustration on the POPRES data [21], but with different choices of weight and data renormalization (see Methods and Discussion).

The proposed method, LAPSTRUCT, has arisen from the idea of studying the geometry of the intrinsic dependence structure of sample populations, which can be creatively regarded as a weighted graph, together with a metric measuring the degree of relatedness for each pair of individuals. The paradigm of the approach is that local infinitesimal structure integrates out global macroscopic structure. Another interpretation to this is to define a random walk on the weighted graph constructed above, with a suitably normalized transition probability between two nodes reflecting their connectivity. Then one can use the top spectrum of the Markov transition matrix to map the data to a lower dimenional Euclidean space. This idea has clear antecedents in earlier work in population genetics (e.g. [5]).

The results on both the Greenish warbler (a ring species) data set [3], [22] and a simulated data set with a spatially correlated population give better approximations to the true population structure than does PCA. Because Laplacian eigenfunctions are generalized harmonic functions, the patterns observed from the PC map on spatially correlated genetic data [3] are also present in the Laplacian eigenmap. Therefore, any hypotheses of historic migration suggested by LAPSTRUCT would require additional evidence before a conclusion is made.

## Results

### Simulation Study A

**Figure 1:**
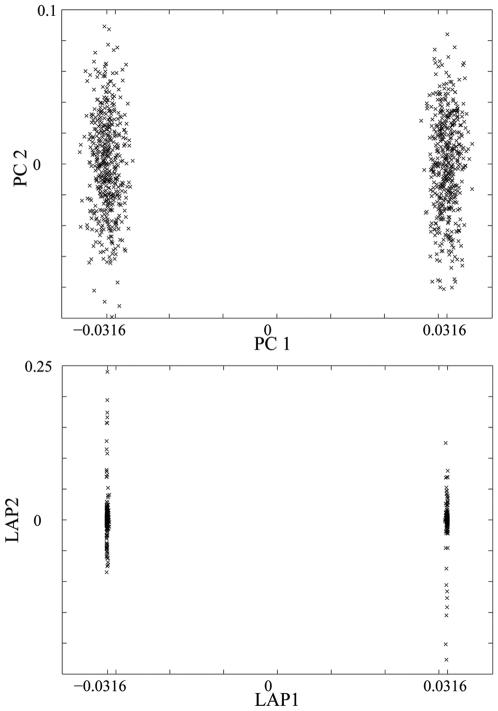
Structure of a simulated discrete population. Population structure detected by PCA (top) and by Laplacian with 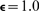 (bottom), for the discrete population consisting of two subpopulations.

**Figure 2:**
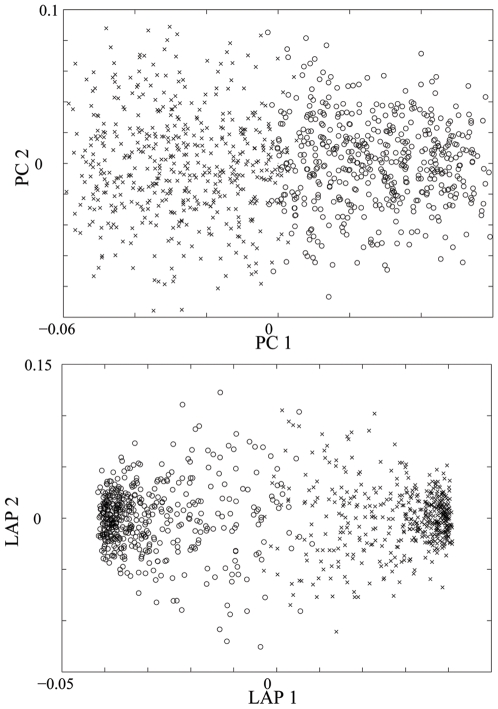
Structure of a simulated admixed population. The ancestral population structure detected by PCA (top) and by Laplacian with 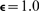 (bottom), for the admixed population with two ancestral populations, where crosses (circles) stand for individuals whose ancestry proportion from ancestral population 1 is larger (smaller) than one-half.

In our simulations, we compare the results of LAPSTRUCT with those of the PC-based method EIGENSTRAT [6]. **Figure 1** illustrates the population structure dectected by EIGENSTRAT and by LAPSTRUCT in the discrete population consisting of two subpopulations (see Methods). In this example, the population structure is perfectly captured by the vector, 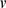, of length 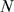, having entry 
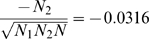
 for each individual in population 1 and entry 
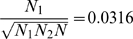
 for each indvidual in population 2, where 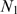 and 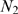 are the total numbers of individuals from subpopulations 1 and 2, respectively, and 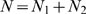 (see **Text** **S1** online for details). Both the PC and the Laplacian eigenvector appear to be approximating 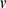, but the Laplacian approach is clearly giving a much more accurate approximation. While both approaches are effective at clustering the data, the more accurate approximation of the ancestry vector, 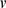, by the Laplacian approach suggests that ancestry should be more accurately accounted for in downstream analyses such as association mapping. In principle, this should increase power, though in our simulation the effect was slight (see **Table 1**). **Figure 2** shows the population structure identified by EIGENSTRAT and by LAPSTRUCT in the admixed population. The PC map shows the expected uniform distribution of ancestry proportion. However, the Laplacian eigenmap shows a tendency to shrink the points toward two clear clusters, indicating the two ancestral populations. For disease association studies conducted in both simulations by simply replacing the PCs by Laplacian eigenfunctions in the regression setting introduced in reference [6], LAPSTRUCT peforms as well as EIGENSTRAT (see **Table 1**).

**Table 1:** Simulated Association Testing.

	EIGENSTRAT	LAPSTRUCT ( 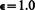 )	LAPSTRUCT ( 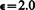 )
Discrete population			
Random SNPs	0.0001	0.0001	0.0001
Differentiated SNPs	0.0001	0.0001	0.0001
Causal SNPs	0.4735	0.4762	0.4739
Admixed population			
Random SNPs	0.0001	0.0001	0.0001
Differentiated SNPs	0.0001	0.0001	0.0001
Causal SNPs	0.4891	0.4919	0.4863

Proportion of association reported as significant by EIGENSTRAT and LAPSTRUCT at significance level 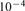, based on 100,000 simulations.

### Simulation Study B

**Figure 3:**
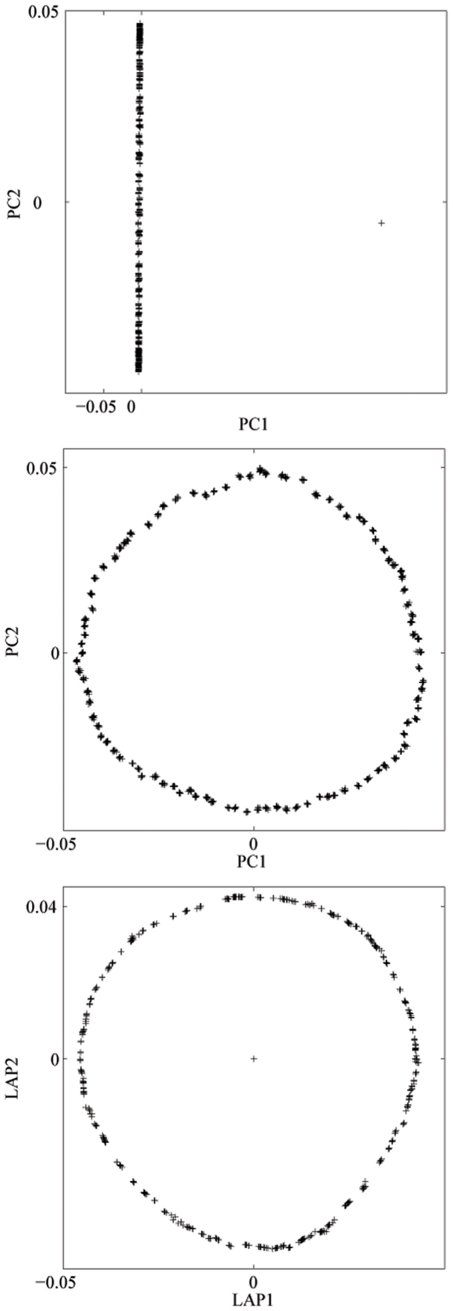
Structure of a simulated ring population. Population structure detected by PCA (with and without outliers present) and by Laplacian with 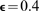, for the simulated ring population.

The sensitivity of PC to outliers is illustrated by the analysis of the spatially correlated population that consists of subpopulations arranged on a circle and an additional isolated subpopulation. When 10 individuals from the isolated subpopulation are included in the sample, the top PC focuses on isolating those outliers, and the PC map based on the top 2 components does not capture the full structure of the data, missing the circle configuration of the population structure (see **Figure 3**). With the outliers removed from the sample, the PC map based on the top two PCs does give the ring shape of the population structure. In contrast, the Laplacian eigenmap based on two components identifies the full population structure even in the presence of outliers, demonstrating that it is much more robust to outliers than is PC. The additional smoothness in the Laplacian eigenmap compared to the PC map might be due to the fact local correlation is weighted more highly, which gives a local smoothing effect.

### Phylloscopus trochiloides

**Figure 4:**
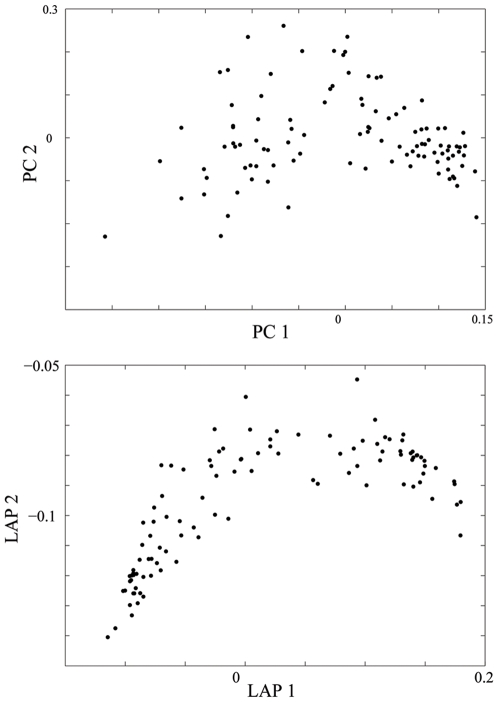
Ring Structure of a real dataset. Population structure detected by PC map and by Laplacian eigenmap with 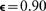, for Greenish Warbler dataset.

**Figure 4** below illustrates the population structure detected by the PCA and Laplacian methods, respectively, where one can more clearly observe the ring-shape structure in the Laplacian eigenmap, compared to the vague structure shown in the PC map.

## Discussion

We have developed LAPSTRUCT, a Laplacian eigenfunction approach for detection and correction of population structure in genetic studies. LAPSTRUCT can be viewed as a robust alternative to PC-based methods such as EIGENSTRAT. Like PC, LAPSTRUCT naturally leads to population clusters according to the degree of genetic correlation among individuals. However, LAPSTRUCT is designed to be less sensitive to outliers than PC, emphasizing structure that affects many data points rather than just a few extreme points. LAPSTRUCT can reveal less noisy and richer structure at different scales by varing the parameters. It is expected to become a promising tool for population genetics.

In the simulation studies, the top Laplacian eigenfunctions identify the overall structure, while the PC approach has a tendency to highlight outliers, when they are present. For example, in the spatial simulation with outliers, PC requires three components to find the ring structure, while the Laplacian eigenfunction approach finds the ring structure with only two components. This suggests that the Laplacian eigenfunction approach could be more useful than the PC approach in contexts such as association mapping in which it is desirable to capture the population structure with as few components as possible, in order to preserve power. Additionally, only those eigenfunctions for which cases and controls have significantly different distributions need to be accounted for in the setting of association mapping, and including unnecessary eigenfunctions will lead to power loss. Further investigation in this direction is encouraged.

The Laplacian eigenmap approach we describe is part of a more general setting of spectrum-based dimension reduction techniques that includes the PC approach. The appropriate choice of the neighborhood parameter, 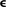, is what causes the Laplacian eigenmap to be less sensitive to outliers than PC. When 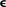 is sufficiently large, the Laplacian eigenmap approach and the PC approach can produce very similar results. As 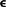 is decreased, the Laplacian eigenmap can capture the local dependence structure at different scales. In practice, 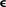 should be chosen reasonably large to make the graph connected and maintain valid type one error for association studies. For example, 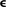 could be the 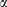-th quantile for some suitable 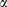. An alternative on the scale of neighborhood is to select each subject's 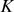 closest neighbors in terms of correlation for some reasonably large integer 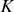. To avoid the issue of tuning parameter selection Lee et al. [20] simply take 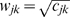 if 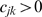, otherwise 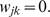 Generally there is room for different choices of weights which may give close performance, and the optimal weight is worth further investigation. The threshholding technique seems appropriate and it has been widely accepted. It reduces the noise from less correlated samples. We incorporate this idea in the renormalization of the genotype data, where each individual's SNP is normalized using the *local* SNP frequency estimated from only those closely correlated individuals. We note this is appropriate when the data are abundant, and one would certainly use all data instead if the sample size were relatively small.

## Materials and Methods

### Phylloscopus Trochiloides (Greenish Warblers) Data

Greenish warblers are most abundant in western and eastern Siberia, where they form a ring species complex. The complex consists of two main populations connected by gene flow via a narrow band of populations to the south that are arranged in a ring around the Tibetan plateau. There is no mating between the two main populations where they overlap geographically, so greenish warblers can be regarded as inhabiting a one-dimensional habitat. Irwin et al. [22] collected 105 individuals from 26 geographic sites and each individual was typed for presence or absence at 62 amplified fragment length polymorphism (AFLP) markers.

### Laplacian Eigenfunctions

Regard each individual 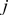 as a vertex 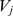 in a weighted graph 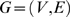, where 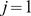 to 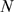. Let the weight between individuals 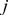 and 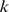 be a Gaussian kernel 
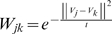
 if 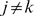 and 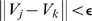, and 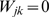 otherwise. Here 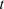 and 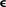 are some selected positive real numbers. The 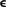 measures the size of each subject's neighborhood. The constant 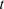 stands for the global diffusion scale on the graph and we set 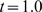 in all the computations. (For information on the effects of 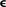 and 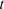 on detection of population structure, see **Figure S1** online.) The 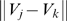 measures the *distance* between vertex 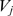 and 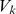. We set the distance 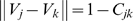, where 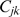 is the estimator of genetic correlation [6] between individuals 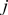 and 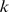. Specifically, let 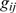 denote the genotype 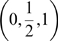 of individual 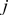 at SNP 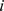. We normalize the vector of genotypes for SNP 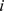 by subtracting off its average, 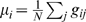, and then dividing each entry by 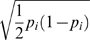, where 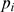 is an estimate of the allele frequency at SNP 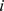 given by 
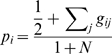
. (All missing entries are excluded from the computation.) Let 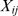 be the resulting normalized genotype for SNP 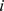 in individual 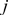. Then we set 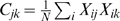.

To avoid the effects of population structure in the allele frequency estimation, the same idea above leads to an alternative local SNP frequency estimation and genotype updating approach. Instead of estimating a single allele frequency per marker, we compute a local SNP frequency 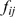 for each individual 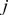 at SNP 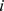 simply by including only those individuals whose correlation with individual 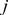 is larger than 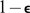. That is, 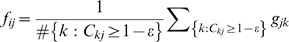. Next we denote the updated genotype matrix G from the original genotype matrix g by 
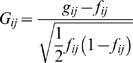
.

Let 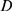 be a diagonal matrix of size 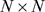 with entries 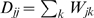, a natural measure on the vertices. The Laplacian matrix on graph 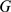 is defined to be 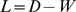. Note that 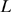 is a symmetric and positive semidefinite matrix, and we restrict to the normalized version 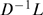 which is not symmetric anymore. The eigenfunctions of the normalized equation 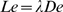 are denoted by 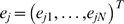 for each 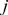, ranked according to the reverse order of their corresponding eigenvalues, i.e., 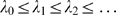. It is easy to see that 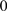 is always an eigenvalue with constant eigenvector consisting of all 1's. These eigenfunctions generalize the low frequency Fourier harmonics on a manifold approximated by the graph 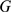. To achieve dimension reduction, the Laplacian eigenmap with first 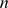 (usually small, 2 or 3) eigenvectors is defined by 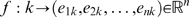 for individual 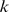. Note that the situation here is different from PCA, where one takes the PCs corresponding to the *largest* eigenvalues which account for the largest amount of variation in the data. The justification is given below. We remark that a symmetrically normalized version of 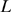 is given by 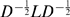. The Laplacian eigenmap using the corresponding spectrum gives comparable performance. For the relationship between these two versions, see [19].

**Figure 5:**
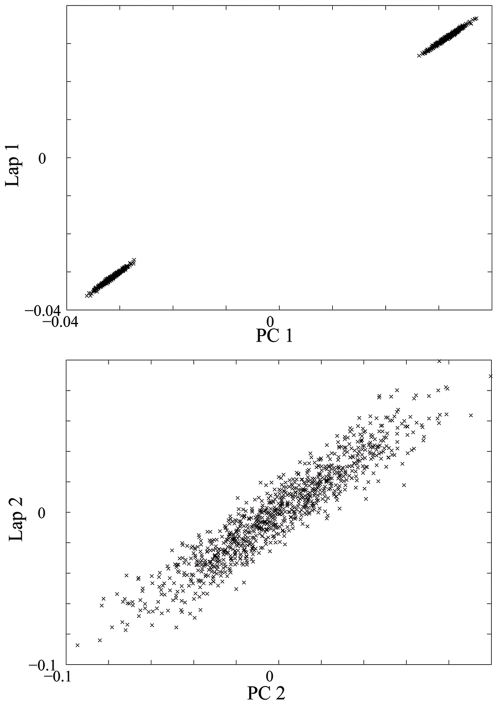
QQ-plot of PCA and Laplacian. QQ-plot of the top two PCs and Laplacian eigenfunctions with 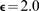 for the simulated discrete population.

The Laplacian eigenmap approach we describe is part of a more general setting of spectrum-based dimension reduction techniques that includes the PC approach. The appropriate choice of the neighborhood parameter, 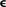, is what causes the Laplacian eigenmap approach to be less sensitive to outliers than PC. When 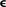 is sufficiently large, the Laplacian eigenmap approach and the PC approach can produce very similar results. This is shown in **Figure 5** for the simulated discrete population model. As 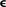 is decreased, the Laplacian eigenmap can capture the local dependence structure at different scales. See **Figure S2** online for an illustration.

To apply the Laplacian eigenmap method to disease association studies, one can follow a multiple regression approach as in [6]. For example, one could regress genotypes and phenotypes on the top 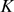 Laplacian eigenvectors for each individual, and then compute the adjusted 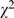 statistic of the residuals. In the simulations, we set 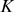 equal to 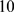, in order to make a comparison with EIGENSTRAT.

### Justification of Weight Kernel and Laplacian Eigenmap

The selected Gaussian weight is optimal in a certain sense, and it has a deep connection to the heat kernel on a manifold that gives the general solution to the heat equation. In the discrete case, the Laplacian of a function can be expressed as combinations of heat kernels which locally approximate the Gaussian kernel. For the mathematical details, see references [15], [17]. The locality preserving property of the Laplacian eigenmap follows from the fact that the cost function of a weighted graph equals the Laplacian of the map function, that is, 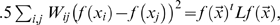 where 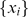 are the collection of nodes and 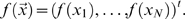 So the minimization problem reduces to finding 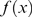 that minimizes 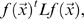 subject to the constraint 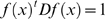, and this is equivalent to the generalized eigenvalue problem stated above. This also explains why the Laplacian eigenmap ranks the eigenvalues in *increasing* order.

### Simulation Study A. Discrete and Admixed Populations

To simulate a discrete population consisting of two subpopulations, we follow a model of population structure used in reference [10] (see also [6]). Each subpopulation is generated by the Balding-Nichols model, but with each subpopulation having its own generalized 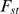 value (0.01 and 0.05, respectively, for subpopulations 1 and 2), instead of the same value for both subpopulations (see [10] for details). The population allele frequency of each random SNP is sampled uniformly from 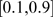. The allele frequency within each subpopulation is drawn from a beta distribution, 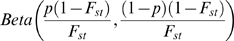. For each individual, 10,000 SNPs were generated. The sample consists of 500 cases and 500 controls, where 60% of cases and 40% of controls were from subpopulation 1 and the rest were sampled from subpopulation 2. For the admixed population with two ancestral populations, the ancestral populations' generalized 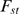 values were set equal to 0.01 and 0.09 respectively. For the admixed population, 1,000 individuals were sampled, half cases and half controls. The sample's ancestral proportions are assumed uniformly distributed from 0 to 1. For the causal allele, a risk model [6] with relative risk 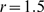 was used for both the discrete population and the admixed population. The allele frequencies for highly differentiated SNPs are respectively set to 0.2 and 0.8 in the two subpopulations.

### Simulation Study B. Spatially Correlated Population

Following reference [3], an equilibrium population is simulated using the software MS for population genetics developed by Hudson [23]. The population consists of 100 subpopulations equally spaced on a circle, with members of an additional isolated subpopulation as outliers. Each subpopulation is assumed to consist of an equal number of diploids. During each generation backward in time, a fraction 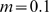 of each subpopulation along the circle is made up of migrants from each adjacent subpopulation, and there are no gamete swaps between non-adjacent subpopulations. 1,000 SNP loci were independently simulated with one segregating site per locus, and 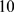 individuals were sampled from each subpopulation.

**URL.** Software for running LAPSTRUCT on a Linux platform is available at http://galton.uchicago.edu/~junzhang/LAPSTRUCT.html.

## Supporting Information

Text S1 Supporting Text
(0.09 MB PDF)

Figure S1 Here we consider the simulated discrete population consisting of two subpopulations, analyzed with ε = 1.0 in all cases. When the scale parameter t is sufficiently small, the Laplacian matrix L degenerates to the identical matrix I and no structure can be detected. When t = 0.1, the second Laplacian eigenfunction degenerates approximately to zero for one of the subpopulations. For larger t values, there are little difference in the detected structures.
(0.08 MB PDF)

Figure S2Here we consider the simulated discrete popualtion consisting of two subpopulations, and t = 1.0 in all cases. When ε = 0.96, the graph has two connected components representing two subpopulations and the top two Laplacian eigenfunctions degenerate to 0 and -1/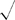 500 = -0.0447. When ε≥1.0, the graph is connected. As ε increases, the local correlation structures revealed by the Laplacian eigenmap evolve to global structures which approximate to PCs.(0.11 MB PDF)
