# Supplementary material for: Laplacian Eigenfunctions Learn Population Structure
Source: PLoS One. 2009 Dec 1;4(12):e7928. doi: 10.1371/journal.pone.0007928 (PMC2779848; doi:10.1371/journal.pone.0007928)
Supplement: Text S1 — Supporting Text (0.09 MB PDF) [file pone.0007928.s001.pdf]

Supplementary Text for:
Laplacian eigenfunctions Learn Population Structure

Jun Zhang[1], Partha Niyogi[2], Mary Sara McPeek[3]

1. Department of Radiology, The University of Chicago, 5841 South Maryland Avenue, Chicago, Illinois 60637

2. Departments of Statistics and Computer Science, The University of Chicago, 5734 South University Avenue, Chicago, Illinois 60637

3. Departments of Statistics and Human Genetics, The University of Chicago, 5734 South University Avenue, Chicago, Illinois 60637

Theoretical ancestry-capturing vector for 2 populations

As part of **Simulation Study A**, we consider a population consisting of two discrete subpopulations, and we compare the results of PCA and LAPSTRUCT to a theoretically-derived ancestry-capturing vector. Here we provide the details on the theoretical ancestry-capturing vector for 2 populations. First note that the matrix C defined in Materials and Methods is orthogonal to the vector with all entries equal to 1, which we call the 1-vector. Therefore the top PC, which is normalized to have length 1, will be orthogonal to the 1-vector. Similarly, as described in Materials and Methods, the 0th eigenvector by the Laplacian approach is the 1-vector, so the 1st Laplacian eigenvector, which is normalized to have length 1, will be orthogonal to the 1-vector. Therefore, the optimal value, from the point of view of capturing ancestry, for the top PC and for the 1st Laplacian eigenvector would be a vector that is orthogonal to the 1-vector, that is of length 1, and that captures the ancestry perfectly, i.e. takes a constant value on population 1 and a different constant value on population 2. It is easy to verify that the only two vectors satisfying these properties are the vectors v and $-v$, where v is of length N and has entry $\frac{-N_2}{\sqrt{N_1 N_2 N}}$ for each individual in population 1 and entry $\frac{N_1}{\sqrt{N_1 N_2 N}}$ for each indvidual in population 2, where N_1 and N_2 are the total numbers of individuals from subpopulations 1 and 2, respectively, and $N = N_1 + N_2$.
