# Supplementary figures and images for: Laplacian Eigenfunctions Learn Population Structure
Source: PLoS One. 2009 Dec 1;4(12):e7928. doi: 10.1371/journal.pone.0007928 (PMC2779848; doi:10.1371/journal.pone.0007928)

Supplementary Figure 1 for:

## Laplacian eigenfunctions Learn Population Structure

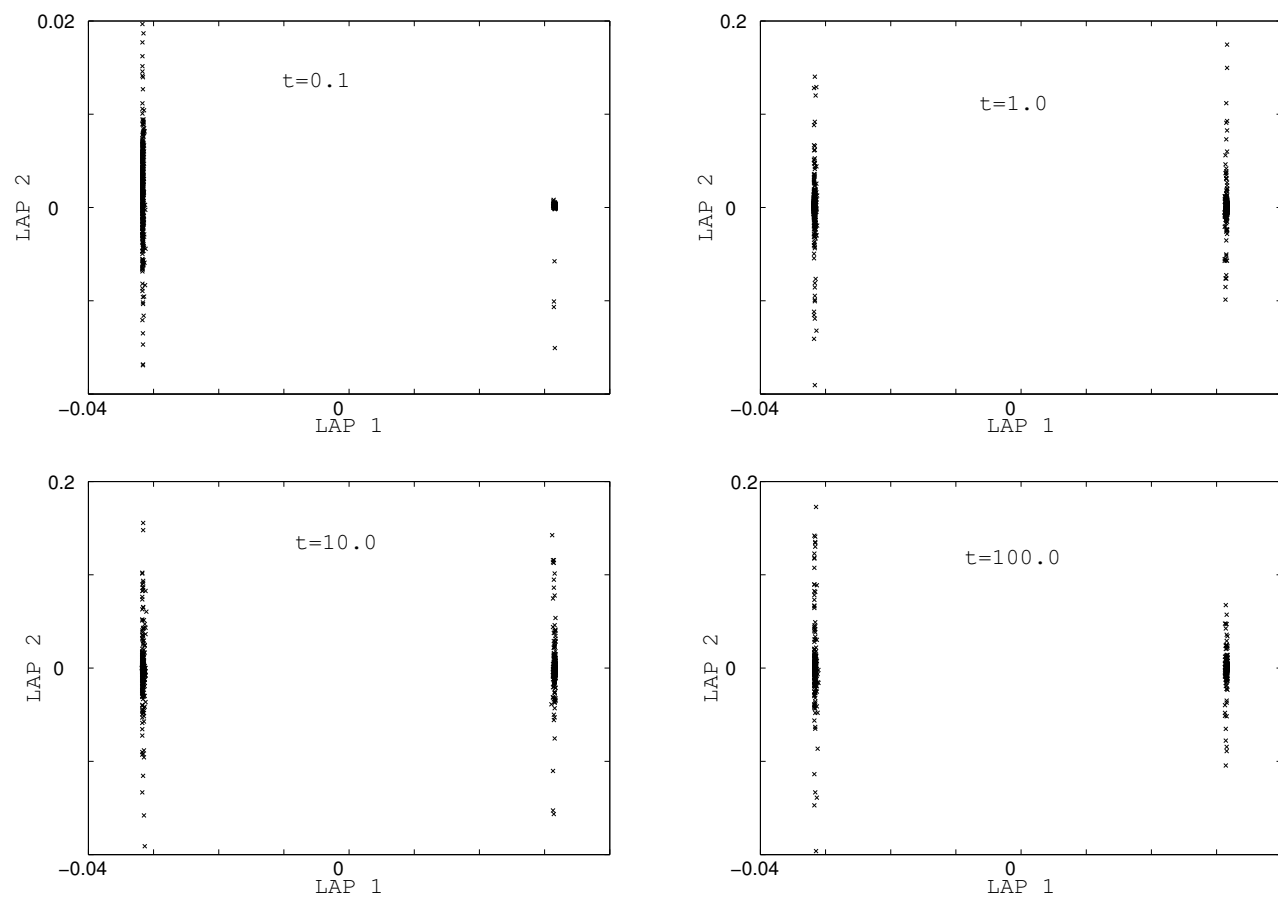

Supplement: Figure S1 — Here we consider the simulated discrete population consisting of two subpopulations, analyzed with ε = 1.0 in all cases. When the scale parameter t is sufficiently small, the Laplacian matrix L degenerates to the identical matrix I and no structure can be detected. When t = 0.1, the second Laplacian eigenfunction degenerates approximately to zero for one of the subpopulations. For larger t values, there are little difference in the detected structures. (0.08 MB PDF) [file pone.0007928.s002.pdf]

Supplementary Figure 2 for:

# Laplacian eigenfunctions Learn Population Structure

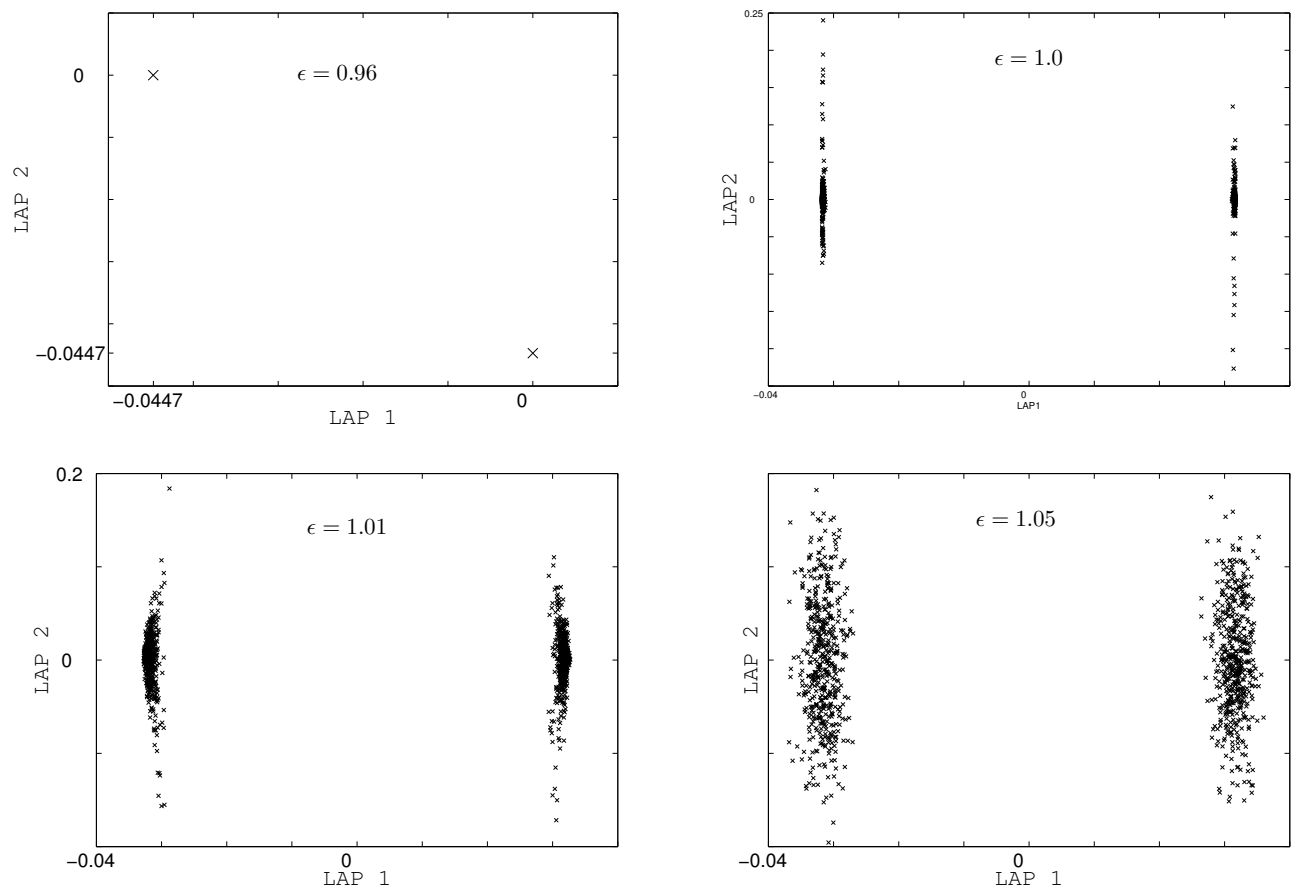

Supplement: Figure S2 — Here we consider the simulated discrete popualtion consisting of two subpopulations, and t = 1.0 in all cases. When ε = 0.96, the graph has two connected components representing two subpopulations and the top two Laplacian eigenfunctions degenerate to 0 and -1/ 500 = -0.0447. When ε≥1.0, the graph is connected. As ε increases, the local correlation structures revealed by the Laplacian eigenmap evolve to global structures which approximate to PCs. (0.11 MB PDF) [file pone.0007928.s003.pdf]
